# Lychee Fruit Detection Based on Monocular Machine Vision in Orchard Environment

**DOI:** 10.3390/s19194091

**Published:** 2019-09-21

**Authors:** Qiwei Guo, Yayong Chen, Yu Tang, Jiajun Zhuang, Yong He, Chaojun Hou, Xuan Chu, Zhenyu Zhong, Shaoming Luo

**Affiliations:** 1 Academy of Contemporary Agricultural Engineering Innovations, Zhongkai University of Agriculture and Engineering, Guangzhou 510225, China; guoqiwei@zhku.edu.cn (Q.G.); chenyayong@zhku.edu.cn (Y.C.); houchaojun@zhku.edu.cn (C.H.); chuxuan@zhku.edu.cn (X.C.); smluo@gdut.edu.cn (S.L.); 2 College of Biosystems Engineering and Food Science, Zhejiang University, Hangzhou 310058, China; yhe@zju.edu.cn; 3 Guangdong Key Laboratory of Modern Control Technology, Guangdong Institute of Intelligent Manufacturing, Guangzhou 510070, China; zy.zhong@giim.ac.cn

**Keywords:** overlapped lychee detection, monocular vision, Hough circle, three-point definite circle, LBP-SVM

## Abstract

Due to the change of illumination environment and overlapping conditions caused by the neighboring fruits and other background objects, the simple application of the traditional machine vision method limits the detection accuracy of lychee fruits in natural orchard environments. Therefore, this research presented a detection method based on monocular machine vision to detect lychee fruits growing in overlapped conditions. Specifically, a combination of contrast limited adaptive histogram equalization (CLAHE), red/blue chromatic mapping, Otsu thresholding and morphology operations were adopted to segment the foreground regions of the lychees. A stepwise method was proposed for extracting individual lychee fruit from the lychee foreground region. The first step in this process was based on the relative position relation of the Hough circle and an equivalent area circle (equal to the area of the potential lychee foreground region) and was designed to distinguish lychee fruits growing in isolated or overlapped states. Then, a process based on the three-point definite circle theorem was performed to extract individual lychee fruits from the foreground regions of overlapped lychee fruit clusters. Finally, to enhance the robustness of the detection method, a local binary pattern support vector machine (LBP-SVM) was adopted to filter out the false positive detections generated by background chaff interferences. The performance of the presented method was evaluated using 485 images captured in a natural lychee orchard in Conghua (Area), Guangzhou. The detection results showed that the recall rate was 86.66%, the precision rate was greater than 87% and the F_1_-score was 87.07%.

## 1. Introduction

Lychees are one of the most popular fruits and are widely cultivated in the hilly regions of southern China [1,2]. In 2018, the average annual yield of lychees in China was approximately 2.87 million tons, which brought considerable profits and tax revenue for lychee growers and the government. Therefore, this abundant annual lychee production signifies the importance of lychee yield estimation technologies. Current estimation methods mainly use satellite or low-altitude remote sensing technology or image information captured by ground-based vehicles, and fruit detection methods for these methods are inevitably adopting machine vision techniques. To date, an extensive amount of image analysis has been performed for the detection of many kinds of fruits [3,4,5,6,7,8].

It is a fundamental task to detect individual fruits for guaranteeing precision agricultural practices and this has become a hot spot in recent studies. These studies indicate that the growth of fruits in a monocular machine image mainly shows two phenomena—occlusion and overlapping—in the orchard environment [8,9,10,11,12]. Specifically, the occlusion phenomenon is caused by leaves and branches and the overlapping phenomenon is caused by neighboring lychee fruits. Xu et al. proposed a segmentation method that used the Snake model and corner detectors for detecting two apples in an overlapped condition; this segmentation method has an average error of 6.41% when detecting two apples in an overlapped condition. However, their method can detect apples in overlapped conditions only when there is no occlusion from branches or leaves [13]. Xiang and Jiang et al. proposed an algorithm based on binocular stereovision to improve the recognition performance for clustered tomatoes; in their method, edge curvature analysis was adopted to generate the edges map for fruits segmentation and achieved the recognition accuracy rate of 87.9%. However, their method needs to sort every curvature of the pixels on the edge, which makes the calculation time dependent on the length of the edge. When detecting clusters with a greater number of fruits carrying a long edge length in the foreground region, the calculation time will increase accordingly [14]. Liu and Zhao et al. adopted color information from both the red/green/blue (RGB) and hue/saturation/intensity (HSI) color space to train a back propagation neural network for apple segmentation; the Euclidean distance was used to calculate the edge area of segmented apples, from which the apples were recognized. The recognition accuracy of apples in a natural environment can be improved using this method. However, a high recognition error rate occurs when apples are overlapping or when a shadow occurs in the captured images [15]. Nguyen et al. proposed a detection method for occluded apples on trees with an RGB 3D camera using a camera system to build both color (RGB) and three-dimensional (3D) shape information of apples on trees. However, this algorithm is sensitive to light conditions and requires light shield construction to block direct sunlight in the testing orchard [16]. Chen et al. proposed a data-driven fruits detection method using a deep learning strategy to recognize apple and orange clusters, and achieved high accuracy. They trained the classifier using the NVIDIA Titan X graphics processing unit, which took more than 50 thousand iterations for the classifier’s convergence. Although the deep learning strategy provides higher performance for detecting fruits, it depends heavily on the applied hardware devices [17]. Recently, Zhuang et al. proposed a detection method using the combination of marker-controlled watershed transform (MCWT) and convex hull operation to locate citrus fruits in overlapped and occluded conditions. However, their algorithm performs an average in detecting lychee clusters; hence, the watershed transform algorithm is sensitive to complex texture disturbances on lychee fruit surfaces [18]. He et al. proposed a method of green lychee recognition that used an improved linear discriminant analysis (LDA) classifier for classifying pixels and Hough transform circle detection to locate the fruit of lychee by the spherical shape features; this method provides a 76.4% recognition accuracy of clustered lychee fruits. However, in their method, the threshold of the Hough circle radius must be calculated by counting the average number of pixels of each lychee in the test sample images before running the Hough transformation. Therefore, their method greatly increases the workload of lychee detection in practical applications [19].

To summarize, the occurrence of background chaff interferences, such as branches, foliage, sky, remains challenging for target fruit detection using machine vision systems. In particular, detection methods for complicated overlapping conditions of neighboring lychee fruits have rarely been reported. Therefore, the overall objective of the research is to detect overlapping conditions of growing lychee fruits in orchard environments with changing illumination. There are three specific objectives of this study: (1) improve the contrast between the lychee fruits and background objects for images captured in environments with changing illumination; (2) extract potential lychees in occluded growing conditions; and (3) filter out some false detections using uniform local binary patterns (LBP) and a histogram intersection kernel (HIK)-based support vector machine (SVM).

## 2. Materials

### 2.1. Datasets

A variety of lychee called “Jing Gang Hong Nuo” was investigated in early to mid-May in Guangzhou. A total of 485 images (called dataset 1, with 2145 lychee fruits in total) and a total of 86 images (called dataset 2, with 213 lychee fruits in total) were captured using the cameras equipped on the iPhone 7 and SAMSUNG S6 mobile phones respectively in a natural lychee orchard, approximately 2–4 weeks before harvest. Due to the different settings of the adopted cameras, the resolution of the images in dataset 1 was 3024 × 4032 pixels while that in dataset 2 was 2560 × 1440 pixels.

**Figure 1:**
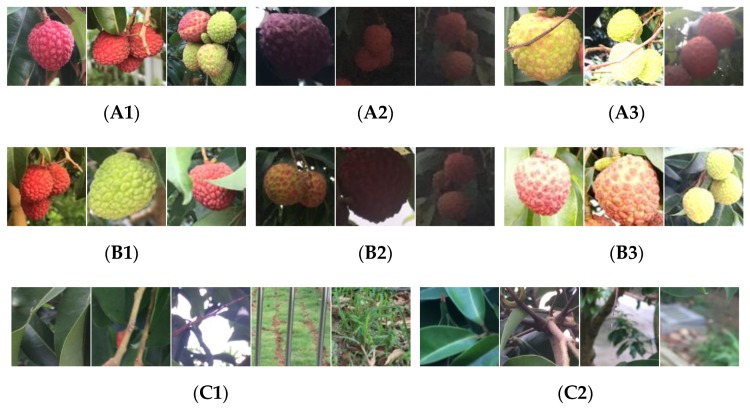
Examples of lychees in an orchard environment with a resolution of 100 × 100 pixels. (**A1**) Well-illuminated examples of dataset 1. (**A2**) Weakly illuminated examples of dataset 1. (**A3**) Overexposure-illuminated examples of dataset 1. (**B1**) Well-illuminated examples of dataset 2. (**B2**) Weakly illuminated examples of dataset 2. (**B3**) Overexposure-illuminated examples of dataset 2. (**C1**) Non-lychee samples of dataset 1. (**C2**) Non-lychee samples of dataset 2.

In addition, the images in both datasets were captured with the cameras located approximately 30–100 cm from the lychee fruits under three different imaging circumstances, including well-illuminated, weakly illuminated and overexposure-illuminated conditions. The number of images captured under well-illuminated, weakly illuminated and overexposure-illuminated conditions were 152, 128 and 205, respectively, in dataset 1; and the number of images captured under the three different conditions were 26, 27 and 33, respectively, in dataset 2. Some examples in both dataset 1 and dataset 2 can be found in Figure 1.

Specifically, 200 images (i.e., 50 images captured under well-illuminated conditions, 50 images under weakly illuminated conditions and 100 images under overexposure-illuminated conditions) were manually selected from dataset 1 and served as the training samples. The lychee fruits in these images were collected as the positive training samples, and the regions containing only background participants were cropped as the negative samples. The remaining 285 images in dataset 1 were taken as test images, and the lychee regions (1604 lychee fruits in total) in the test images were manually annotated and served as the ground truth, as Figure 1(A1–A3) show. All images of dataset 2 were used as testing data, whose examples of which are shown in Figure 1(B1–B3). Figure 1(C1,C2) show non-lychee samples of foliage, branch, sky, pathway and lawn conditions of dataset 1 and dataset 2.

### 2.2. Application Hardware Architecture Design

**Figure 2:**
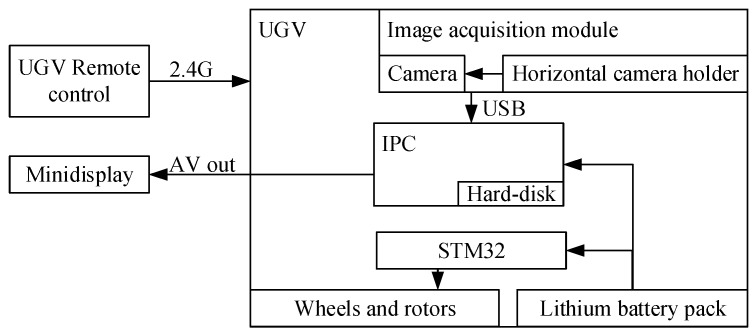
Structure diagram of the unmanned ground vehicle (UGV) system.

At present, the estimation methods mainly use satellite remote sensing technology or image information captured by unmanned ground vehicles (UGVs) [20] or unmanned aerial vehicles (UAVs) [21], the fruit detection methods of which are inevitably adopting computer vision techniques. This paper takes camera mounted UGVs as an example. The camera mounted UGV system consisted of remote control, image acquisition module, lithium battery pack and industrial personal computer (IPC). The 2.4 GHz network motion signal of the UGV was sent by a remote control to the vehicle-mounted microcontroller STM32 to realize wheel and motor control. The UGV was equipped with an IPC powered by lithium battery pack, connected to an image acquisition module via USB. The angle of the camera on the module could be adjusted by the horizontal camera holder, and the acquired images were sent to the hard disk of the IPC for storage through USB. The image was processed by detection algorithm on the IPC, and the processed results were output to the mini-display by AV signal. The structure diagram of the UGV system is shown in Figure 2.

## 3. Lychee Fruit Detection Method

**Figure 3:**
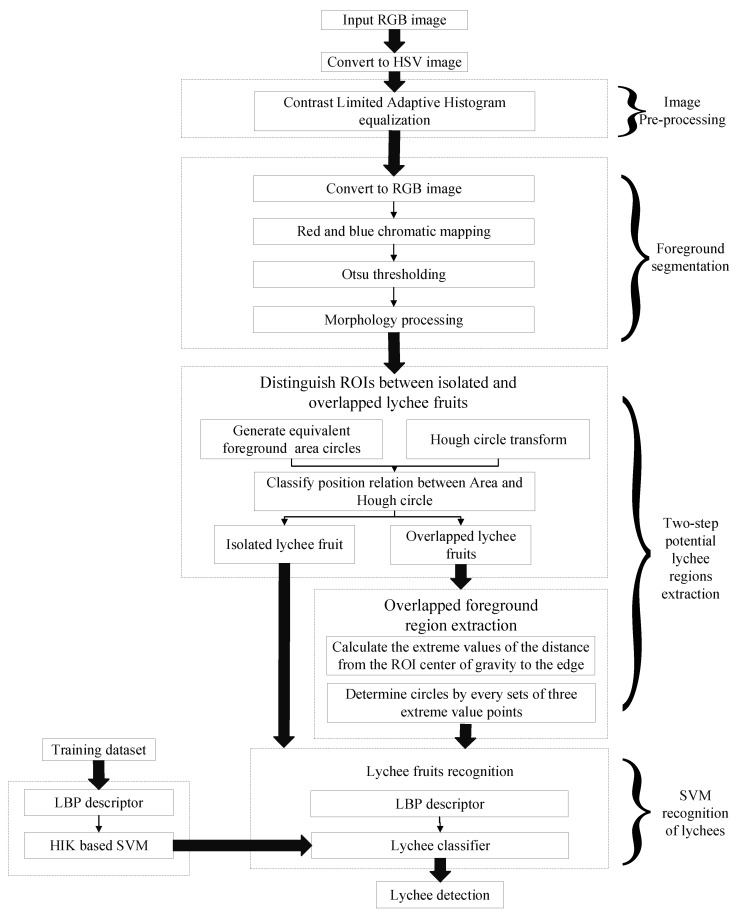
Flow diagram of the proposed lychee detection method.

Figure 3 shows a block diagram that summarizes the key procedures of the proposed lychee detection method, including image preprocessing, foreground segmentation, potential lychee region extraction and status identification.

### 3.1. Image Preprocessing and Foreground Segmentation

Due to the randomness of the location and lighting conditions of the naturally growing lychees, the color space of the imaging results can be distorted by changing the light angle and position. It is was necessary to pre-process the images using an illumination compensation procedure, which focused on adjusting the images captured under weak and overexposed illumination. The foreground segmentation procedure was used for segmenting the foreground and the background of lychee images, which improved the accuracy of the further processes. The image preprocessing and foreground segmentation preliminarily extracted the regions of interest (ROIs).

#### 3.1.1. Image Preprocessing

Since the RGB image data could be easily affected by the illumination conditions in the orchard, the collected lychee images were prone to weak or overexposed illumination. Therefore, it was necessary to adjust the illumination distribution of the resultant images. To avoid altering the hue and saturation information, the input image was first converted from the RGB color space to the hue/saturation/value (HSV) color space, and the intensity component V was selected for illumination adjustment using the contrast limited adaptive histogram equalization (CLAHE) algorithm. There are two main parameters involved in the CLAHE algorithm [22], the number of blocks and the contrast enhancement limit, which are determined by trial and error method. These parameters were selected as 8 × 8 and 0.02, respectively, in the CLAHE algorithm for apple detection in an orchard environment [23]. In this study, in order to determine the setting of the number of blocks, different settings including 5 × 5, 8 × 8, 10 × 10, 12 × 12 and 15 × 15 were considered; for contrast enhancement limits, 0.005, 0.01, 0.015, 0.02 and 0.025 were included. In order to obtain the optimal parameter combination, 10 lychee images in the training dataset 1 were randomly selected, using the CLAHE algorithm under 25 groups of different parameters (5 numbers of blocks × 5 contrast enhancement limits). After processing with the R-B chromatic method for lychee fruit foreground segmentation, the average relative overlap rate *S* of each set of parameters was calculated using following equation:

(1) $$s=\frac{area\left(R_{h}\cap R_{r}\right)}{area\left(R_{h}\cup R_{r}\right)}.$$

Here, *R_h_* represents the segmented region after using the CLAHE algorithm and $R_{r}$ represents the manually segmented standard region for reference. As the results show in Table 1, when the number of blocks was 10 × 10 and the contrast enhancement limit was 0.015, the average relative overlap rate reached the highest value, 86%. Therefore, 10 × 10 and 0.015 were selected as the parameters to be used for the CLAHE algorithm in this paper.

After the processing using the CLAHE algorithm, the illumination adjusted intensity component V’ was obtained; then the three components including the H, S and V’ were used to convert back into RGB color space. The lychee images from the test dataset were used to evaluate the performance of the above procedure.

**Figure 4:**
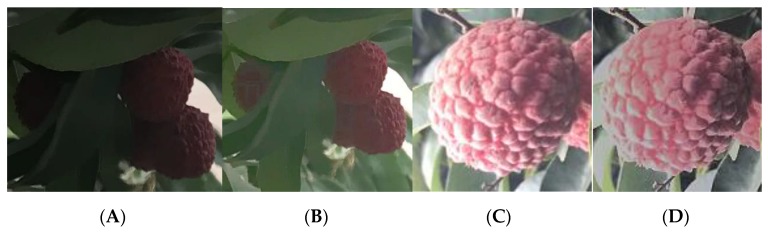
Effect of lychee image illumination compensation. (**A**) Weakly illuminated lychees. (**B**) Weakly illuminated lychees after illumination compensation. (**C**) Overexposed lychees. (**D**) Overexposed lychees after illumination compensation.

The image illumination compensation method for the weakly illuminated and overexposed lychees in the images is shown in Figure 4. Figure 4A,C show images captured under weakly illuminated and overexposure illumination conditions, respectively. Figure 4B,D correspond to Figure 4A,D respectively, after processing with the CLAHE method. More detail is obtainable in Figure 4B than in Figure 4A since the weak illumination is compensated by the preprocessing method. This method also adjusts the overexposed regions, thereby providing more detailed information about the lychee fruits, as shown in Figure 4C,D.

#### 3.1.2. Lychee Foreground Segmentation

**Figure 5:**
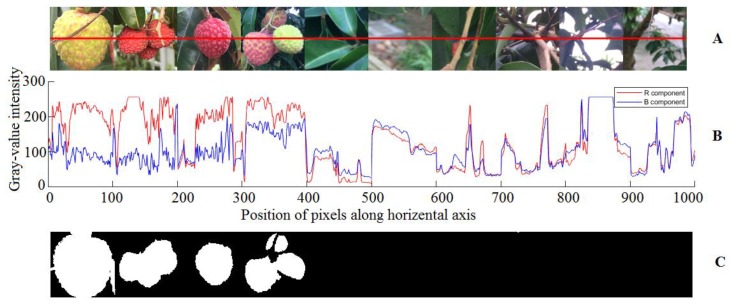
RGB color component statistics of lychees, immature lychees and background. (**A**) Example diagrams and sample red line. (**B**) The R and B component curves corresponding to the sampling red line. (**C**) Results of R-B chromatic mapping with morphological algorithm.

Currently, lychee foreground segmentation is generally processed by the chromatic aberration method combined with an image processing algorithm. For example, Xiong et al. separated mature lychee from orchard background by dividing the Cr component threshold of YCbCr space [24], and Zhuang et al. [18] realized the segmentation of yellow citrus by using the method based on R-G chromatic mapping. However, these two methods are both not ideal for the segmentation of green immature lychee, which occurs often in our datasets. To efficiently separate and identify lychee regions in the image and exclude background, this study extracted information of lychee and background and performed a statistical analysis of the RGB color space. From any horizontal scan line across lychee fruits and background, as shown in Figure 5A, the color intensities of the pixels within the lychee fruit regions, wherein the position pixels along the horizontal axis are between 0 and 400, were different from those within the background regions, wherein the position pixels along the horizontal axis are between 400 and 1000.

Specifically, in RGB color space, the grey-level intensity of the pixels within the lychee region in the red component R was always much higher than that in the blue component B, as shown in Figure 5B (the pixels along the horizontal axis between 0 and 400). In contrast, the grey-level intensity of pixels within the background region in the component R was always approximately equal to that in the component B. Therefore, the lychee fruits could be separated from the background region using red and blue (R-B) chromatic mapping. The segmentation result is shown in Figure 5C, which shows that the R-B chromatic mapping is an appropriate method.

In conclusion, the R-B chromatic subtraction method in RGB color space was adopted in this paper, which is especially suitable for foreground segmentation of lychee images under complex orchard environment conditions. To obtain more stable results, R-B was changed to a relative value as (R − B)/B. Then, the Otsu image segmentation algorithm was used to extract the potential fruit regions [25]. The combination of mathematical morphology methodology including erosion, dilation and hole filling operations were used to remove some image noise, bridge weakly connected potential fruits and fill holes, respectively, in the resultant binary image obtained by the Otsu algorithm.

**Figure 6:**
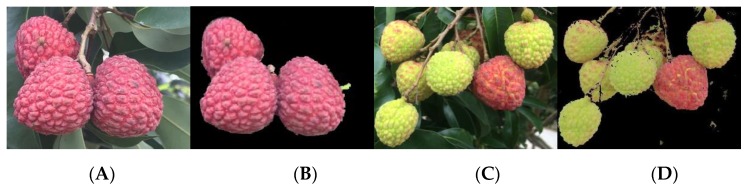
Performance evaluation of lychee foreground segmentation. (**A**) Original image of a lychee cluster, which is labelled I_1_. (**B**) Foreground segmentation result of I_1_. (**C**) Original image of a lychee cluster, which is labelled I_2_. (**D**) Foreground segmentation result of I_2_.

After the foreground enhancement processing, the lychee image was further processed by R-B color component difference, in which chromatic aberration is the result that is statistically analyzed in the color space components. The difference between the foreground and background was further enlarged by increasing the contrast, and the Otsu algorithm was used for foreground segmentation. Figure 6 shows the foreground segmentation results. The results show that the image basically meets the requirement of complete foreground segmentation in the background segmentation process.

### 3.2. Two-Step Potential Lychee Region Extraction

To increase the lychee detection accuracy, this paper analyzes the position state of each lychee through topological analysis and determines the position state of each lychee so that the lychees in a simple state can be easily detected. Moreover, further separation processing was performed for the overlapping areas of complex lychee adhesion.

#### 3.2.1. Distinguishing the ROIs between Isolated and Overlapped Lychee Fruits

The images of the lychee fruits in the orchard could be roughly divided into three statuses: single isolated status, occluded (covered with leaves and branches) status, and overlapped status. Moreover, the differences in lychee fruit shape, which are not perfect circles or ovals, are complicated. Therefore, when using only the Hough circle method to transform the contour of a lychee, the obtained results often have deviations. Based on the above discussion, this paper proposes a status determination method for lychee positions (isolated, occluded, overlapped) based on the Hough circle and a lychee equivalent foreground area circle to eliminate the deviations in the Hough circle judging method. Here, the equivalent foreground area circle by definition is a circle that has the equal size of area to the lychee foreground region, and the center of the circle is the center of gravity of the lychee foreground area. Different from the Hough circle generation by edge information, the equivalent foreground area circle is determined by the position and area of each separate foreground area. This equivalent foreground area circle can accurately represent the location and total size of the potential lychee area after segmentation. The Hough circle refers to the circle generated by Hough circle transformation according to the boundary of the lychee fruit in the segmented foreground lychee image, which can roughly approximate the circle shape of the boundary of the potential lychee region. By analyzing the relationship between the Hough circle and the equivalent foreground area circle, it can quickly and accurately locate the position and status of a lychee in an image. Therefore, this paper proposes a lychee state judgment method based on equivalent area circles and Hough circles (ACHC), and its operating steps are as follows:

**Input:** Binary images of lychee fruits foreground after segmentation *I_f_*.

**Step 1.** Select every foreground region *C_n_* from *I_f_* one by one. (*n* = 1, 2, …, *N*, *N* is the number of lychee regions)

**Step 2.** Extract the edge of *C_n_* and operate the Hough transform.

**Step 3.** Calculate the circle center *Oh_n,i_*(*X_n,i_*, *Y_n,i_*) and radius *Rh_n,i_* of each Hough circle. (i = 1, 2, …, *p*, *p* represents the number of Hough circles generated in *C_n_*)

**Step 4.** Generate the equivalent foreground area circles of *C_n_* by calculating its circle center *Oa_n,j_*(*X_n,j_*, *Y_n,j_*) and radius *Ra_n,j_*. (j = 1, 2, …, *q*, *q* represents the number of equivalent foreground area circles in *C_n_*)

Here, the coordinates of the equivalent foreground area circle center *Oa_n,j_*(*X_n,j_*, *Y_n,j_*) equal the coordinates of the center of gravity in *C_n_*, the radius *Ra_n,j_* is found using Equation (2):

(2) $$Ra_{n,j}=\sqrt{area\left(C_{n}\right)/\pi },$$

where the operation area() is to calculate the area.

**Step 5**. Calculate lychee fruits status *S_n_* of *C_n_* using Equations (3)–(8), where *S_n_* = 1 means single isolated, *S_n_* = 2 means occluded, *S_n_* = 3 means overlapped status.

**Figure 7:**
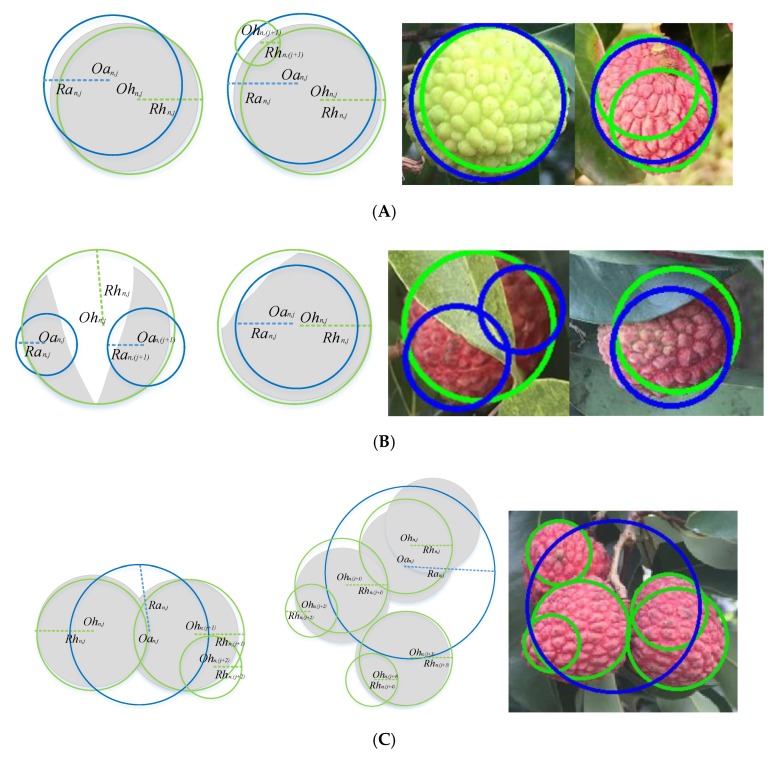
Topological diagrams of the lychee location relationships. (**A**) Single isolated status. (**B**) Occluded (covered with leaves and branches) status. (**C**) Overlapped status.

Topological diagrams of the lychee location relationships are shown in Figure 7 below, where the grey area represents the *C_n_*, the green circles represent the Hough circles *Oh_n,i_* of *C_n_* and the blue circles represent the equivalent foreground area circles *Oa_n,j_* of *C_n_*.

Figure 7A shows that when the lychee foreground image is in a single isolated status, the position and shape size of the Hough circle is similar to that of the equivalent foreground area circle. Assuming that the Hough circle radius is *Rh_n,j_*, the equivalent foreground area circle radius is *R_an_*, the number of equivalent foreground area circles is p, and the number of Hough circles is q, then the following expressions can be obtained:

(3) p=1  and  q≥1,

(4) $$max(Rh_{n,i})\approx max(Ra_{n,j})$$

where the operation max() is to select the maximum value in an array, therefore, $max(Rh_{n,i})$ and $max(Ra_{n,j})$ respectively represent the maximum radius of Hough circles and equivalent foreground area circles in the same foreground region *C_n_*.

Figure 7B shows that when the lychee foreground image is in an occluded status, the following expressions can be obtained:

(5) $$\{\begin{array}{c} p>1\;and\;q=1\\ max(Rh_{n,i})\gg max(Ra_{n,j}) \end{array}$$

(6) $$\sum \left(\pi {\left(Rh_{n,i}\right)}^{2}\right)<\pi {\left(Ra_{n,j}\right)}^{2}$$

Figure 7C shows that when the lychee foreground image is in an overlapped status, the following expressions can be obtained:

(7) $$\{\begin{array}{c} p=1\;and\;q>1\\ max(Rh_{n,i})\ll max(Ra_{n,j}) \end{array}$$

(8) $$\pi {\left(Ra_{n,j}\right)}^{2}<\sum \left(\pi {\left(Rh_{n,i}\right)}^{2}\right)$$

**Output:** Lychee fruits status *S_n_*.

#### 3.2.2. Individual Fruit Extraction from Overlapped Lychee Regions

Lychee fruits often grow in clusters, which leads to overlapped regions in the images. In our team’s previous work, we used the watershed transform method and convex hull operation to solve slight overlapping problems in the extraction of citrus fruit clusters, and this approach achieved accurate and efficient performance. However, the segmentation procedure using watershed transform might be affected by the changes in the noisy gradient; thus, this method might suffer from oversegmentation and generate many small foreground regions, which would further blur the contours of the segmented foreground objects. Unfortunately, the texture and color components on the surface of a lychee are much more complicated than those of a citrus fruit, which causes many kinds of noise in the image. Therefore, an extreme value segmentation method based on transverse searching in polar coordinates is proposed (PCEVP); this method can reduce the amount of computation and overcome the problem of noise interference in lychee fruit images. The algorithm steps run as follows:

**Input:** The foreground region *C_n_* contains overlapping lychee fruits determined by ACHC (*S_n_* = *3*).

**Figure 8:**
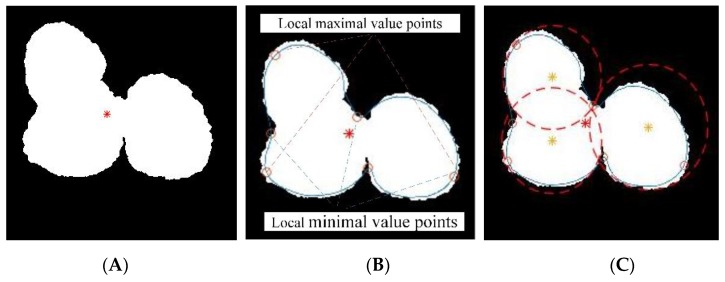

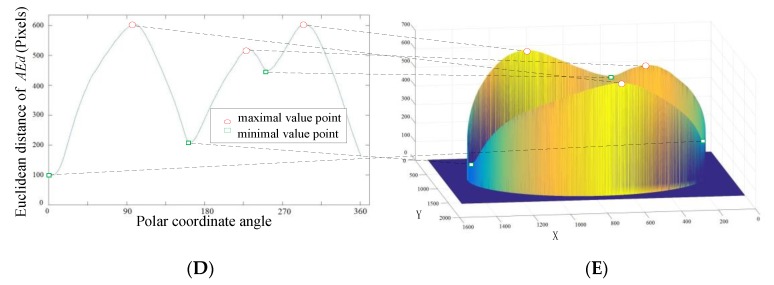
Example of image processing using polar coordinate extreme value projection (PCEVP) processing. (**A**) Determine the center of gravity of the foreground region (**B**) by calculating all local maximal and minimal value points on the boundary of the foreground region. (**C**) Individual foreground regions in (**B**) segmented using PCEVP. (**D**) The Euclidean distance $\left|AE_{d}\right|$ and extreme value points of the foreground region boundary. (**E**) The Euclidean distance $\left|AE_{d}\right|$ in bar graph in a 3D coordinate system.

**Step 1.** Extract the edge *E* of *C_n_* and the center of gravity *A* in *C_n_*, as shown in Figure 8A.

**Step 2.** Take the center of gravity A as the origin of the polar coordinate system.

**Figure 9:**
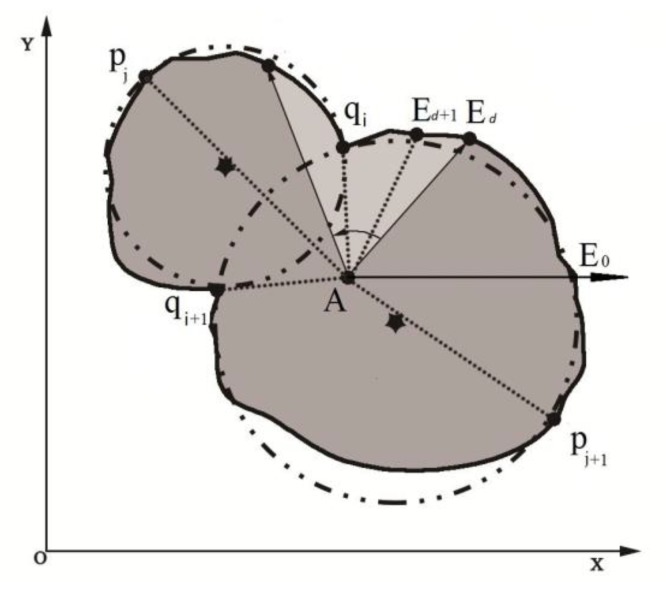
Geometric calculation model of the extreme value points of the boundary of two lychees in the foreground region.

**Step 3.** Calculate the distance from the origin A to the pixel points (indexed by *d*) on the edge of the domain using Equation (9) for every degree (360°) in a counter-clockwise direction, as shown in Figure 8B.

(9) $$\left|AE_{d}\right|=\sqrt{{\left(x_{Ed}-x_{A}\right)}^{2}+{\left(y_{Ed}-y_{A}\right)}^{2}}$$

where the coordinates of *A* and *E_d_* in the XOY coordinate system are $A\left(x_{A},y_{A}\right)$ and $E_{d}\left(x_{Ed},y_{Ed}\right)$, respectively. Figure 9 shows an example of a geometric calculation model of two overlapping lychee fruits.

**Step 4.** Calculate every maximal value point *p_j_* and minimal value point *q_i_* on the edge E (*i* = 1, 2, …, *m*, *m* is the number of minimal value points; *j* = 1, 2, …, *m*, *m* is the number of maximal value points).

**Step 5.** Separate the edge *E* by “*q_i_*-*p_j_*-*q*_*i*+1_” order, as shown in Figure 8B (“*q_i_*-*p_j_*-*q*_*i*+1_” represents “local minimal value point - local maximal value point - local minimal value point” order).

**Step 6**. Determine circles by every set of three extreme points “*q_i_*-*p_j_*-*q*_*i*+1_”, as shown in Figure 8C.

A large amount of image data shows that overlapping in the lychee fruit foreground region is a common phenomenon [26,27]. Therefore, PCEVP was used to further segment the overlapping foreground regions. Figure 8A shows the center of gravity of the foreground region (marked by a red asterisk) using mathematical morphology operations. Figure 8B indicates that the extreme value points can be found by calculating a graph of the Euclidean distance of the boundary of the foreground region, and Figure 8E shows the extreme value points in a 3D coordinate system. Figure 8D shows a graph of the Euclidean distance and extreme value points of the boundary of the foreground region. The local maximal and minimal value points always exist in the following order: “local minimal value point, local maximal value point, local minimal value point”. Figure 8E shows the result of calculating all local maximal and minimal value points on the boundary of the foreground region, and the extracted individual foreground objects are marked with red dotted circles in Figure 8C, which are determined circles by every set of three extreme points: “local minimal value point, local maximal value point, local minimal value point”.

**Output:** Location and size of every single lychee fruit in the foreground region *C_n_*.

### 3.3. LBP-SVM Recognition of Lychee Fruit

**Figure 10:**
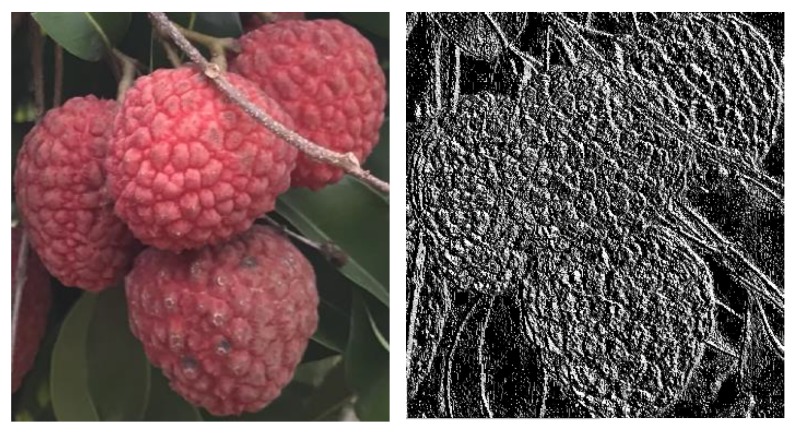
Local binary pattern (LBP) operator representation of a lychee image.

To improve the reliability of lychee detection results, a local binary pattern based support vector machine (LBP-SVM) classifier was adopted for recognition after data sample training. A uniform local binary pattern (LBP) is a parameter-less operator used to describe the local structure features of an image. An LBP operator is an image texture descriptor, while the surface texture of the lychee and the texture of the background have great differences that can be recognized by the naked eye. The image data represented by an LBP operator is of great significance to the perception and recognition of lychees [28]. The lychee image data are represented by LBP, as shown in Figure 10.

By defining a linear optimal hyperplane, the classification problem is transformed into an optimization problem to determine the hyperplane; note that the HIK-based SVM is approximately 2000 times faster than the general nonlinear kernel SVM [29]. When $F_{1}$ and $F_{2}$ are two histogram features of image data defined with the LBP, the HIK-based SVM function $K\left(F_{1},F_{2}\right)$ expression is as follows:

(10) $$K\left(F_{1},F_{2}\right)=\sum_{l=1}^{n+1}\min\left[F_{1}\left(l\right),F_{2}\left(l\right)\right]$$

Under the condition of image data acquisition in this paper, the imaging distance was 30–100 cm. Within our 2145 lychee fruit samples, the average number of pixels in the 160 smallest samples was 861 and the average number of pixels in the 160 largest samples was 1578. Therefore, to filter out the image artefacts with either small or large pixels while maintaining as many as possible potential fruit regions, only the ROIs with sizes between 800 and 1700 pixels were fed into the SVM model.

## 4. Results and Discussion

The performance of the proposed method was evaluated using the dataset described in Section 2. All the experiments were conducted based on MathWorks MATLAB R2018a.

### 4.1. Performance Evaluation under Well-Illuminated Conditions without Using LBP-SVM Classifier

**Figure 11:**
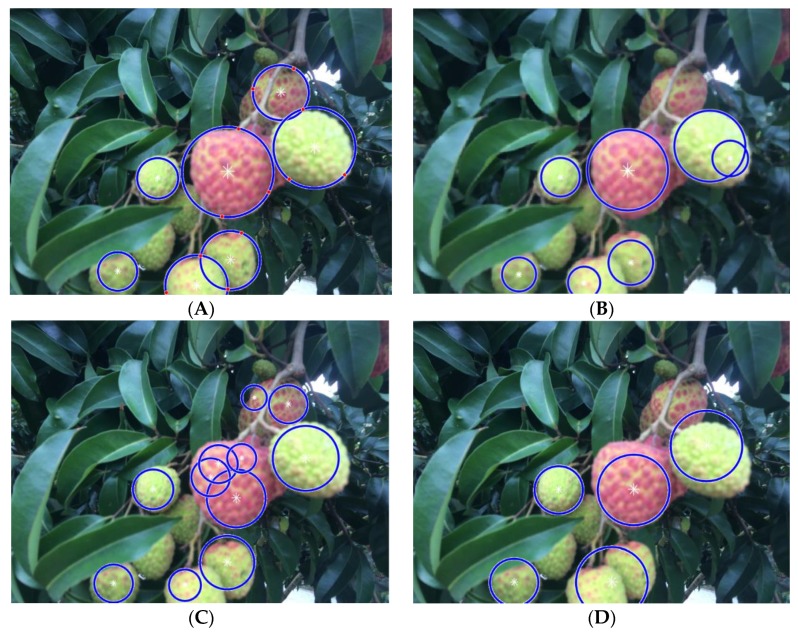
Results of lychee fruit cluster detection performance under well-illuminated conditions without using the local binary pattern based support vector machine (LBP-SVM) classifier. (**A**) Results of the proposed Method A. (**B**) Results of Method B. (**C**) Results of Method C. (**D**) Results of Method D.

To evaluate the performance of the two-step potential lychee region extraction method, well-illuminated lychee fruit images were first selected from test datasets A1 and B1, as shown in Figure 11. The use of the proposed two-step potential lychee region extraction method was called Method A. When Method A was replaced with the Hough transform circle detection method [19] in our detection framework, the above integrated procedures formed Method B. When Method A was replaced with the watershed transform [18] in our detection framework, the above integrated procedures formed Method C. When Method A was replaced with the sampling pixels on edge region methods that Liu and Zhao proposed [15] to segment lychee fruits in our detection framework, the above integrated procedures formed Method D.

Figure 11 shows the results of lychee fruit cluster detection performance under well-illuminated conditions without using the LBP-SVM classifier by Method A, Method B, Method C and Method D. Figure 11A shows that lychee fruit clusters can be nicely detected (the blue circles fit more closely around the edges of the lychee fruits) under well-illuminated conditions using Method A. In the following figures, the blue circles represent the local result areas of the lychee fruits detected by the above methods. The red dots on the edge of the blue circle represent the extreme points of the lychee region determined by the PCEVP method proposed in this paper. The basic principle of the PCEVP method is to detect the potential lychee fruit region using the above red dots based on the “local minimum value point, local maximum value point, local minimum value point” arrangement rule and the three-point definite circle theorem.

Figure 11B shows the results of Method B for comparison, which indicates that under the same test picture and the same preprocessing conditions, the results of the Hough circle test may have both missed fruits and made repetitive detections. The Hough circle transform (Method B) generally exhibited deficiencies in the following two aspects: (1) the region of the detected Hough circle in the image is generally smaller than the real region of a lychee; (2) two or more Hough circles are easily generated by mistake for the same lychee. Here, the reason for the first deficiency is that in the detection of the edge of the foreground region of lychee fruits after segmentation under various illuminated conditions, due to the irregular shape of the foreground region of the lychee fruits, the area detected by the Hough circle will be smaller than the real area of lychee fruits. The reason for the second deficiency is that the data images used in this paper were taken at a distance of 30–100 cm from the lychee fruits, which resulted in a large range of lychee fruit sizes in the test data. Therefore, the range setting of the Hough circle radius threshold was also large, thereby increasing the rate of repetitive detection via Hough circle transformation.

Figure 11C shows the results using Method C for comparison, where the watershed transform algorithm was adopted. The results show that oversegmentation occurred using watershed transform. When conducting the watershed transform, to guarantee accurate segmentation of foreground objects, the ideal situation is to consider the foreground regions as a topographic surface and the lowest point of the topographic surface as the catchment basin. However, the watershed transfer might be sensitive to some disturbances that affect the characteristic of topographic surface of lychee fruits, such as significant texture appearance of lychee fruits, environment illumination, camera angle and camera noise, resulting in oversegmentation of lychee fruit regions.

Figure 11D shows the results using Method D for comparison, where the sampling pixels on edge region method [15] was adopted. The results show that Method D is more suitable for the identification of lychees in single isolated status with a faster recognition speed, but its segmentation accuracy of overlapping lychee fruits is not high. Through the analysis of the operation process of Method D, it is found that Method D can only identify one lychee fruit for each independent lychee fruit foreground region, which leads to its low accuracy in identifying overlapping lychee fruits. In order to improve the accuracy of overlapping lychee fruits of Method D, we conducted an additional corrosion morphology operation on the foreground area of overlapping lychee fruits detected before its operation, which improved the accuracy of overlapping lychee fruits of Method D to a certain extent.

Here, TP (true positive) is the number of correctly detected lychee fruits, FN (false negative) is the number of lychee fruits missed in detection, FP (false positive) is the number of background participants that were misclassified as lychee fruits, Precision is the proportion of TP to all positive examples (TP + FP), where P = TP/(TP + FP), Recall (recall rate) refers to the proportion of the number of true detections to the total number of lychee fruits, where Rc = TP/(TP + FN). To further evaluate the performance of the methods, the F_1_-score is used to combine the metrics, including the number of TPs, the number of FPs and the number of FNs. The F_1_-score is adopted and defined as follows:

(11) $$F_{1}-score=2\frac{P\cdot Rc}{P+Rc}.$$

The values of the F_1_-score for the different methods are also shown in Table 2. The results also demonstrate that the proposed Method A is more appropriate. Table 2 shows that: (1) The proposed method achieved acceptable detection results in both test datasets, where the detection performance is similar. Among them, their recall rate is around 89%, accuracy rate is around 80% and F_1_-score is close to 84%, indicating that proposed Method A is not sensitive to different settings of image acquisition procedure and has a certain generalization ability. (2) The detection speed is the average detection time (seconds per frame) for the test dataset. The time consumption of Method A in detecting images with lychee fruits is around 1 s, which is a little higher than the average time consumption of the other three methods (Method B is 0.745 s, Method C is 0.821 s, Method D is 0.654 s). (3) The test results of Method C indicate that it had the highest in recall rate, 93.02%, in three methods, which is about 4%–5% higher than the other two methods, but the testing precision rate of Method C is only 68.38%, which is caused by the oversegmentation phenomenon of watershed transform algorithm, and thus produces far more numbers of FP results than the other two methods. (4) The test results of Method D indicate that it had the fastest average detection time. The amount of FP (false positive) results is similar to Method A and Method B in dataset A1. The recall rate of Method D is 80.34%.

This result indicates that the method proposed in this paper can be used to detect clusters of lychee fruits and individually extract each fruit. The results also show that the proposed Method A can avoid misjudging a single lychee under occluded conditions by using ACHC processing, which can divide the potential foreground region of lychee fruits into three cases—isolated, occluded and overlapped—without any omission.

### 4.2. Performance Evaluation under Overexposure and Weakly Illuminated Conditions without Using the LBP-SVM Classifier

**Figure 12:**
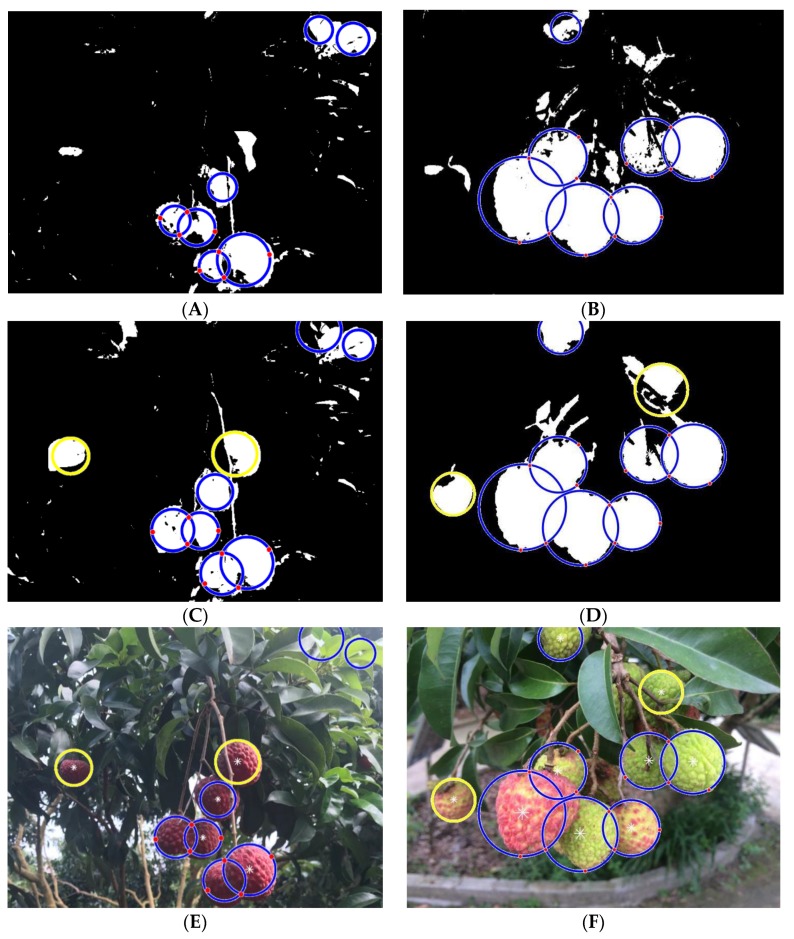
Results of isolated and lychee fruit cluster detection performance under overexposure and weakly illuminated conditions without using the LBP-SVM classifier. (**A**,**B**) Foreground segment results without using image preprocessing. (**C**,**D**) Foreground segment results using image preprocessing. (**E**,**F**) Results of lychee fruit detection using Method A.

Due to the variable lighting conditions in the orchard environment, the collected images will be overexposed and weakly illuminated. In view of the above situation, the image preprocessing step described in Section 3.1.1 is added to the method. The purpose of this test is to evaluate the influence of the preprocessing method on the precision and recall rate of the proposed detection method. Performance evaluation under overexposure and weakly illuminated conditions without using the LBP-SVM classifier is shown in Figure 12.

Figure 12A,B represent the foreground segmentation and the final lychee extraction results, respectively, of the proposed Method A without image preprocessing in two different images of clustered lychee fruits from test datasets A2, A3, B2 and B3. Figure 12A,B, which do not use image preprocessing, contain many more white holes in the foreground than Figure 12C, which uses image preprocessing; this finding indicates that the foreground of two weakly illuminated lychee fruits is not successfully segmented in Figure 12A. Similarly, comparing Figure 12B,D shows that the foreground of two overexposed lychee fruits is not successfully segmented in Figure 12B. Figure 12C,D show that after using the image preprocessing method, the segmented lychee fruit foreground areas under either weakly illuminated or overexposed conditions are enhanced, which enables the successful detection of lychee fruits in weakly illuminated conditions, as shown by the yellow circles in Figure 12E,F. Figure 12 shows that image preprocessing based on the CLAHE algorithm improves the illuminated conditions by transforming each pixel with a transformation function derived from a neighborhood region, and uses the contrast enhancement limit to remove the effect of noise. Thus, the performance of the proposed method was improved by such preprocessing procedure.

Table 3 shows that the time consumption of the proposed Method A in detecting overlapped lychee fruits under overexposed and weakly illuminated conditions is 1.242 s, which is nearly 0.2 s longer than the time consumption of the proposed Method A in well-illuminated conditions. When detecting lychee fruits under overexposed conditions using test data containing 634 lychee fruits in 214 pictures, among which the number of correct detections (TP) is 540, the number of missed detections (FN) is 94, the number of incorrect detections (FP) from background interference is 126, the precision rate reaches 81.08% and the recall rate is 85.17%. When detecting lychee fruits under weakly illuminated conditions, the test data included 268 lychee fruits in 94 images, the number of TPs is 227, the number of FNs is 41, the number of FPs is 67, the precision rate reaches 77.21% and the recall rate is 84.07%. In the end, the comprehensive precision of this method without the SVM classifier under overexposure and weakly illuminated conditions is 79.90%, the recall rate is 85.03% and the F_1_-score 82.38%. In contrast, as the sample shows in Figure 12E,F, the recall rate of Method B is 82.48% with a precision rate of 76.23%.

The results show that the method proposed in this paper can detect isolated, occluded and overlapped lychee fruits growing in various orchard environments, such as cloudy, sunny days, overexposed or uneven brightness conditions. However, there are always different interferences in an orchard environment, which can increase the probability of system misjudgment and will reduce the robustness of the whole detection system. Therefore, the detection results using the LBP-SVM classifier are tested and discussed below.

### 4.3. Performance Evaluation in an Orchard Environment Using the LBP-SVM Classifier

**Figure 13:**
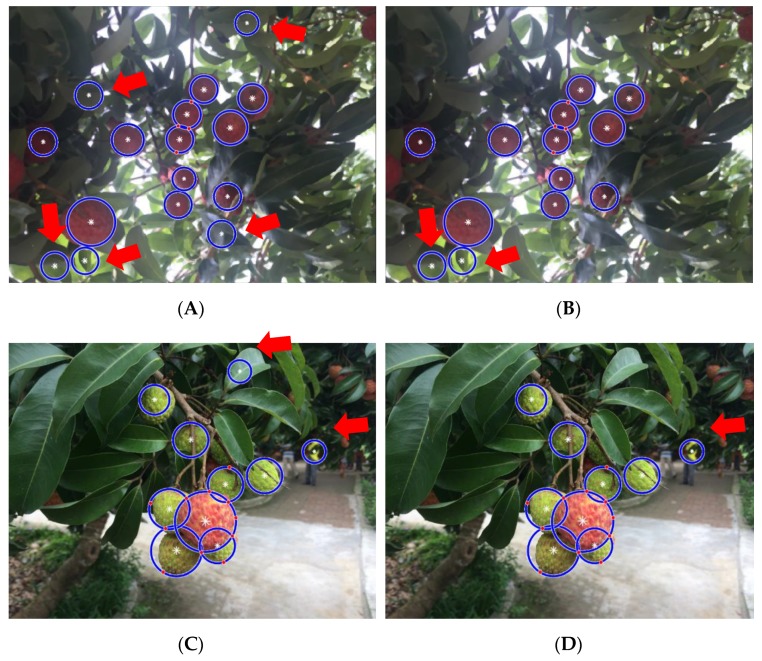
Lychee fruit recognition results in orchard environments using the LBP-SVM classifier. (**A**,**C**) Results without using the LBP-SVM classifier. (**B**,**D**) Results using the LBP-SVM classifier.

The purpose of this test is to evaluate the performance of the LBP-SVM classifier in filtering out the FP detections generated by the background chaff interferences and calculate the improvement in the detection precision rate provided by the LBP-SVM classifier for the detection method proposed in this paper. The test results are shown in Figure 13.

To evaluate the performance of the LBP-SVM module, 568 lychee fruit and 277 background target samples were extracted from the training images to train the SVM classifiers using HIK, linear and nonlinear kernel functions. The remaining samples served as the test samples. Figure 13 shows that isolated and overlapped lychee fruits could be detected under variable illumination conditions using the LBP-SVM classifier, thereby reducing the FN rate. Figure 13A,C indicate that the lychee fruit cluster can be detected, and the red arrows indicate seven FP detection errors, wherein the leaves—as background interference—are mistakenly detected as immature lychees. Figure 13B,D show that four FP errors are removed by the LBP-SVM classifier. Herein, there are three FP errors still in Figure 13B,D, which are indicated by three red arrows, in which two are caused by misjudging the leaves and the other is caused by misjudging a person wearing a green jacket. Figure 13 indicates that the misjudgment caused by leaves was eliminated by the SVM classifier. However, the misjudgment caused by the person wearing a green jacket has not been eliminated because this occasional background interference was not added to the training data set of the SVM classifier.

According to the detection results shown in Table 4, the TP rate was 86.69%. The FP rate was 13.34%. The FN rate was 12.40%. The precision, recall and F_1_-score were 87.48%, 86.66% and 87.07%, respectively. The highest TP rate was 88.75%, which was achieved under well-illuminated conditions. The lowest TP rate was 84.70%, which was under weakly illuminated conditions. The time consumed by the proposed Method A mainly occurred during clustered lychee extraction and clustered lychee matching. The time consumed by the isolated lychee detection mainly occurred in the searching of the fruit regions using the LBP-SVM classifiers, and the total average processing time was nearly 0.2 s longer than that without using the LBP-SVM classifier. The average time consumed from the extraction of clustered overlapped lychee fruits to fruit localization was 1.412 s. However, adopting the LBP-SVM classifier greatly reduced the number of FPs—from 126 down to 80—under overexposure illumination, which increased the precision rate from 81.08% to 87.10%. Similarly, the LBP-SVM classifier reduced the number of FPs from 67 down to 38 under weakly illuminated conditions, and the precision rate correspondingly increased from 77.21% to 85.66%. Moreover, the LBP-SVM classifier reduced the number of FPs from 151 down to 81 under well-illuminated conditions, and the precision rate subsequently increased from 80.49% to 88.49%. The LBP-SVM classifier improved the recall rate of detection by approximately 7%.

To detect and match lychee fruits and clusters under natural orchard environments, the key detection method proposed in this paper uses ACHC processing to identify and locate the isolated lychee fruits. This processing approach can avoid the disadvantages of mistaken matches in single lychees under occluded conditions and simultaneously distinguish the lychee cluster for further detection. After ACHC processing, a clustered lychee fruit matching method based on a three-point definite circle theorem was proposed, which was referred to as PCEVP processing. Specifically, PCEVP processing is designed for identifying and locating adhesion and overlapping of lychee fruits. Finally, to reduce the probability of an FN of lychee fruits, the LBP + SVM classification method was applied. The lychee fruits in the orchard environment with changing illumination conditions were further tested by incorporating the recognition results of the LBP + SVM classifiers. The results illustrate that the method could accurately identify the clustered lychee fruits from complicated backgrounds. The performance of the procedure by incorporating fruit recognition indicates that the proposed Method A could account for the robustness against the influence of occlusion and variable illumination to some extent. The above results indicate that the proposed detection method could more accurately detect single lychee fruits than overlapped lychee clusters. From the interactive performance of the proposed Method A using the test dataset, the average processing time from the extraction of clustered lychees was 1.412 s, which could meet the requirements of automatic yield estimation.

The demonstrated performance shows that the proposed Method A could detect lychee fruits and clusters successfully and robustly in an orchard. However, there are still some shortcomings of the proposed method A. Firstly, the color and texture information of the acquired lychee color images will inevitably change when the illumination intensity in the orchard changes dramatically. Although the training samples used to train the LBP-SVM classifiers contained the information of three different kinds of illumination and textures, the resultant classifier could still not cover all the possible illumination conditions; therefore, some parts of the pixels may also be incorrectly classified. Secondly, when most of the clustered lychee fruits were occluded, some seriously overlapped clustered lychee fruits may be incorrectly matched; hence, further research is needed to improve the matching accuracy of seriously overlapped clustered lychee fruits in orchard environments.

## 5. Conclusions

This study proposed a two-step detection method for lychee clusters using monocular machine vision technology for yield estimation in orchard environments. An effective algorithm was initially given to solve complicated overlapped and occluded conditions of lychee fruits. The main conclusions are as follows:

(1) The proposed two-step lychee fruits detection method can detect clusters of lychee fruits under well-illuminated conditions using different image acquisition equipment. This method can avoid misjudgments in detecting single lychees under occluded conditions when using ACHC processing. Overlapped lychee fruits can be further separated using PCEVP processing, which is helpful to avoid missed detections. The results show that the comprehensive precision of this method without SVM classifier in well-illuminated conditions is 80.49%, the recall rate is 88.75% and the F_1_-score is 84.42%.

(2) The two-step method can also be adopted to detect lychee fruits under overexposed and weakly illuminated conditions with a recall rate of 85.03% and an F_1_-score of 82.38%. However, without using the LBP-SVM classifier, the precision rate is only 79.9% as a result of misjudging background chaff interferences. Furthermore, the results demonstrated that the proposed method can be used for fruits in different levels of maturity, including lychee fruits.

(3) The misjudged detection results can be filtered out by the LBP-SVM classifier, which helps reduce the FN rate and provides a precision rate of 87.48% and a recall rate of 86.66%, which is nearly 8% higher than that without the use of the LBP-SVM classifier. An average running time of 1.412 s using the test lychee images was recorded in the experiments, indicating that the proposed method has obtained real-time performance for lychee detection in natural environment.

Further improvements will be considered by incorporating more effective descriptors for lychee detection with higher precision rates and exploring an improved PCEVP method for lychee fruits growing in heavy and dense clusters.

## Figures and Tables

**Table 1:** The average relative overlap rate *S* of 25 sets of parameters used in contrast limited adaptive histogram equalization (CLAHE).

The Contrast Enhancement Limit	The Number of Blocks				
	5 × 5	8 × 8	10 × 10	12 × 12	15 × 15
0.005	78%	79%	80%	80%	77%
0.01	78%	81%	83%	80%	78%
0.015	79%	84%	86%	82%	80%
0.02	79%	81%	85%	82%	82%
0.025	78%	79%	82%	80%	81%

**Table 2:** Detection results of overlapped lychees under well-illuminated conditions without using the LBP-SVM classifier.

Method	Test Dataset	Average Detection Time (s)	Total Lychee Fruits	TP	FN	FP	Precision (%)	Recall (%)	F_1_-Score (%)
A	A1	1.081	702	623	79	151	80.49	88.75	84.42
A	B1	0.994	213	190	23	50	79.17	89.20	83.89
B	A1	0.745	702	611	91	177	77.54	87.04	82.01
C	A1	0.821	702	653	49	302	68.38	93.02	78.82
D	A1	0.654	702	564	138	162	77.69	80.34	78.99

**Table 3:** Detection results of overlapped lychees under weak or overexposure illumination without using the LBP-SVM classifier.

Method	Illumination State	Average Detection Time (s)	Total Lychee Fruits	TP	FN	FP	Precision (%)	Recall (%)	F_1_-Score (%)
A	Weak	1.226	634	540	94	126	81.08	85.17	83.08
A	Overexposure	1.261	268	227	41	67	77.21	84.70	80.78
A	Weak and overexposure	1.242	902	767	135	193	79.90	85.03	82.38

**Table 4:** Lychee fruit detection results in an orchard environment using the LBP-SVM classifier.

Illumination Conditions	Average Detection Time (s)	Lychee Fruits	TP	FN	FP	Precision (%)	Recall (%)	F_1_-Score (%)
Weak	1.42	634	540	94	80	87.10	85.17	86.12
Overexposure	1.42	268	227	41	38	85.66	84.70	85.18
Well	1.38	702	623	79	81	88.49	88.75	88.62
Comprehensive	1.41	1604	1390	214	199	87.48	86.66	87.07

## Abbreviations

ACHC	Equivalent foreground area circles and Hough circles
CLAHE	Contrast limited adaptive histogram equalization
HIK-SVM	Histogram intersection kernel based support vector machine
HSI	Hue/saturation/intensity color space
HSV	Hue/saturation/value color space
IPC	Industrial personal computer
LBP	Local binary pattern
LDA	Linear discriminant analysis
PCEVP	Polar coordinate extreme value projection
RGB	Red/green/blue color space
ROI	Region of interest
UAV	Unmanned aerial vehicle
UGV	Unmanned ground vehicle
YIQ	YIQ color space, where Y is Luminance, I is in-phase, and Q is quadrature
YCbCr	Luma/Blue chromaticity component/Red chromaticity component
